# The effects of hydroalcoholic extract of *Nigella sativa seed* on oxidative stress in hippocampus of STZ-induced diabetic rats

**Published:** 2015

**Authors:** Abbasali Abbasnezhad, Parichehr Hayatdavoudi, Saeed Niazmand, Maryam Mahmoudabady

**Affiliations:** 1 *Department of Physiology, School of Medicine, Mashhad University of Medical Sciences, Mashhad, Iran*; 2 *Neurogenic Inflammation Research Center, School of Medicine, Mashhad University of Medical Sciences, Mashhad, Iran*

**Keywords:** *Diabetes mellitus*, *Nigella sativa*, *Oxidative stress*, *Hippocampus*, *Rat*

## Abstract

**Objective:**

Oxidative stress plays an important role in the etiology of diabetic complications. Diabetes impairs hippocampus neurogenesis, synaptic plasticity, and learning. The aim of this study was to investigate the effects of hydroalcoholic extract of *Nigella sativa* seed on oxidative stress in STZ-induced diabetic rats' hippocampus.

**Materials and Methods:**

Diabetes induced by 60 mg/kg STZ, i.p, and the rats were divided into five experimental groups (n=8-10 in each group) including control (received 0.5 ml normal saline), untreated STZ-diabetic (received 0.5 ml normal saline), and treated rats received *Nigella sativa* extract (200 and 400 mg/kg) or metformin (300 mg/kg) by gavage for 42 days. Serum glucose concentration and body weight as well as hippocampus tissue malondialdehyde and thiol levels were determined by calorimetric assay.

**Results:**

Serum glucose level in the diabetic rats treated with 200 mg/kg *Nigella sativa* extract at the days 24 and 45 decreased in comparison to untreated diabetic group (p<0.05, p<0.01, respectively). Weight loss was significantly different between metformin and *Nigella sativa* extract at the dose of 200 and 400 mg/kg (p<0.05). Thiol content of hippocampus increased by 200 mg/kg *Nigella sativa* extract in comparison to untreated diabetic group (p<0.05). Malondialdehyde content of hippocampus reduced by *Nigella sativa* extract, 200 mg/kg (p<0.001), 400 mg/kg (p<0.05), and metformin (p<0.05) in comparison to the untreated diabetic group.

**Conclusion:**

The results of the present study showed that hydroalcoholic extract of the Nigella sativa decreased oxidative stress in hippocampus of the STZ-induced diabetic rats. Nigella sativa at the dose of 200 mg/kg was more effective to reduce oxidative stress in hippocampus of rats.

## Introduction

Diabetes mellitus is a common metabolic illness that is accompanied by high blood glucose concentration as a result of the lack of insulin or the presence of insulin resistance in peripheral tissues or both (momin et al., 2013 ▶; Dalia and Hafez, 2013 ▶). the complications of diabetes are major health problems in developing and developed countries, (Dalia and Hafez, 2013 ▶). Cerebral complications of diabetes are primarily due to the direct effects of chronic hyperglycemia. The hippocampus is involved in learning and memory and is particularly vulnerable to alterations in cerebral glucose supply. Hippocampal neurogenesis, synaptic plasticity, and learning are impaired in the diabetic rats (Amin et al., 2013 ▶; Stranahan et al., 2008 ▶). Diabetes in rats leads to reorganization of hippocampal synapses and dendrites. Moreover, glucocorticoid reactivity is increased in response to stress (Magariños and McEwen, 2000 ▶). Furthermore, low corticosterone levels can prevent the impairment of learning and memory in diabetes (Stranahan et al., 2008 ▶). According to magnetic imaging resonance (MRI) studies, even in well- controlled diabetic patients the hippocampus is destroyed (Yin et al., 2013 ▶).

High blood glucose concentration induces oxidative stress (Capellini et al., 2010 ▶). Free radicals possess one or more unpaired electrons in their outer electronic orbits. ROS (reactive oxygen species) such as superoxide anion (O^2−^), hydroxyl radical (OH^−^), hydrogen peroxide (H_2_O_2_), and singlet oxygen (1O_2_) are highly reactive. Free radicals and ROS are produced in normal physiological processes (Leong et al., 2013 ▶). It is already known that oxidative stress occurs when there is an imbalance between ROS production and antioxidant defense system (Ceretta et al., 2012 ▶). Therefore, oxidative stress plays a key role in the etiology of the diabetes and its complications (Kanter et al., 2004). 

Nigella sativa is a plant from the Ranunculaceae family (Ahmad et al., 2013 ▶). The seeds contain 36–38% fixed oils, 0.4–2.5% essential (volatile) oil, proteins, alkaloids, and saponins (Ali and Blunden, 2003 ▶). The Nigella sativa is well- known for its potent antioxidative effects (Leong et al., 2013 ▶), hence it can protect the brain from the oxidative stress following lipid peroxidation in transient global ischemia of the brain (Azzubaidi et al. 2012 ▶).

Moreover, Nigella sativa can prevent the impairment of spatial memory after scopolamine administration and reducing the AChE (acetylcholinesterase) activity as well as oxidative stress of the brain tissue in rats (Mohammadpour et al., 2013 ▶). 

Therefore, the present study was designed to investigate the effects of a hydroalcoholic extract of the Nigella saliva seed on the oxidative stress due to the STZ-induced diabetes in the hippocampus of rats.

## Materials and Methods

**Plant material and preparation of the extract**

The Nigella sativa seeds were purchased from a local herbal shop in Mashhad, Khorasan province, Iran and identified by botanists in the herbarium of the Ferdowsi University of Mashhad (voucher No. 176-2013-9). The seeds were powdered and soaked in 2 L of a hydroalcoholic solution (50% ethanol, 50% water) for 48 h at room temperature. The extraction solution was subsequently filtered and dried using an oven at 40 °C for 72 h. The dried extract was dissolved in the distilled water to obtain the doses of 200 and 400 mg/kg.

**Chemicals and drugs**

All chemicals were of analytical grade. Streptozotocin (STZ) was obtained from Sigma (Germany). Serum glucose concentrations were determined using Pars Azmun kits (Tehran, Iran) by a photometer

**Animals and induction of diabetes**

Male Wistar rats (250–280 g, 10 weeks old) were housed on a 12 h light-dark cycle, under constant temperature (22±1 ^o^C) and free access to standard laboratory diet and drinking water. All experiments were performed under the license of the Ethics Committee of Mashhad University of Medical Sciences (MUMS) according to the standards of caring and using of the experimental animals.

To induce diabetes, streptozotocin (60 mg/kg, i.p.) was injected at a single dose . We confirmed the development of the diabetes by measuring the blood glucose levels in tail blood samples of the 12 h fasted.rats. The rats with the blood glucose level≥250 mg/dl were considered diabetic. 

**Experimental design**

The rats were randomly assigned to six groups (n=8-10 in each group): control (C), diabetic (D), diabetic-metformin (DM), diabetic-extract (DE). The C and D groups received normal saline, DM group received metformin (300 mg/kg) and DE groups (DE-200 and DE-400) received the Nigella sativa seed extract (200 and 400 mg/kg) by a gavege tube for 6 weeks.

**Preparation of rat** **hippocampus** **tissue**

At the end of the experiment, the animals were anesthetized deeply with ether and euthanized by decapitation with a guillotine. The Hippocampus was rapidly dissected out on ice and stored at -80 °C. The hippocampus samples were homogenized in ice-cold KCl (150 mM) for determination of malondialdehyde (MDA) and thiol levels

**Malondialdeyde (** **MDA)** **assays**

MDA level is an index of lipid peroxidation. MDA reacts with thiobarbituric acid (TBA) as a TBA reactive substance (TBARS) and produces a red complex.

Briefly, 1 ml of the homogenate was added to the 2 ml of a solution containing TBA/trichloroacetic acid (TCA) /hydrochloric acid) (HCL), and then it was boiled in a water bath for 40 minutes. The solution remained to reach to the room temperature, then it was centrifuged at 1000 g for 10 minutes. The absorbance of the supernatant was read at 535 nm (Sharma, 2006 ▶). The MDA concentration was calculated according to the following equation. 

MDA concentration (M) = Absorbance / (1.56 × 10^5^ cm^-1^ M^-1^)

The MDA levels are expressed per gram of tissue.

**Thiol** **assays**

DTNB (2,2'-dinitro-5,5'-dithiodibenzoic acid) reagent, which reacts with the SH group, was used to determine the total thiol groups. The produced yellow complex has a peak absorbance at 412 nm. Briefly, 50 μL of tissue homogenates was added to 1 ml Tris-EDTA) ethylene diaminetetraacetic acid) buffer (pH=8.6) and the absorbance was read at 412 nm against Tris-EDTA buffer alone (A_1_). Then, 20 µl of a 10 mM solution of DTNB was mixed with the solution and stored in the room temperature for 15 minutes and the absorbance was read again (A_2_). The absorbance of the DTNB reagent was also read as blank (B) (Sharma JB, 2006 ▶). The thiol levels were determined using a spectrophotometric method based on the use of Ellman’s reagent (DTNB solution) and the results were expressed per gram of tissue. 

Total thiol concentration (mM) = (A_2_ – A_1_ – B) × 1.07) / 0.05 × 14.150)

**Plasma glucose concentration**

Serum fasting blood glucose concentrations were measured in four different time periods: before STZ injection, at the third day, 24 days after STZ injection (when the diabetes was confirmed), and at the sixth weeks (45^th^ day).

**Data analysis**

The Results are expressed as mean±SEM. Statistical analyses were performed using the one-way ANOVA followed by the Tukey’s test, and statistical significance was defined as p<0.05. 

## Results

The serum glucose concentration increased in days 3, 24, and 45 in the D group compared to the C group (p<0.001), while it didn’t show significant difference between DM and D group in the same days. On the other hand, serum glucose concentration reduced at the days 24 and 45 in the DE-200 compared to the D group (p<0.05, p<0.01, respectively) (Table 1). 

**Figure 1:**
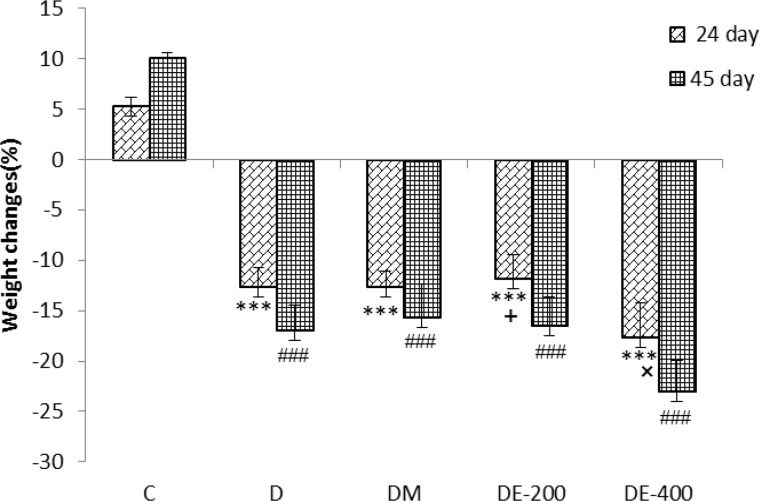
The effect of Nigella sativa extract on the prevention of weight loss in the STZ-induced diabetic rats. n=8 in D group and n=10 in all other groups. ^***^ p<0.001 compared with C group after 24 days. ^### ^p<0.001 compared with C group after 45 days. ^+^ p<0.05 comparison between DE-400 and DE-200 groups in 24 days. ^×^ p<0.05 comparison between DM and DE-400 groups after 24 days. Statistical analyses were made using the one-way ANOVA followed by the Tukey’s test

According to the present results, the weight was increased in the C group (p<0.001) and decreased in the D (p<0.01) and treated diabetic groups (p<0.05) compared to the day zero. Also, in the untreated and treated diabetic groups, the weight was reduced in the days 24 and 45 compared to the control group (p<0.001).But, there was not any significant difference between the untreated diabetic group and treated diabetic groups. However, within treatment groups, 400 mg/kg of the extract was the most effective dose in reducing the weight (p<0.05) (Figure 1).

**Figure 2:**
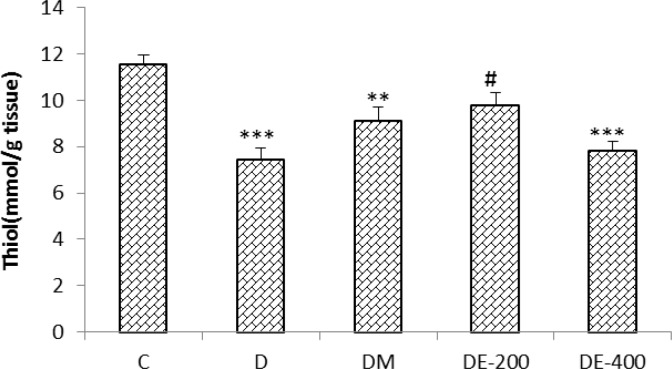
The effect of Nigella sativa extract on the thiol concentration in the hippocampus of the STZ-induced diabetic rats. n=8 in the untreated diabetic group and n=10 in all other groups. ^**^ p<0.01 and ^***^ p<0.001 compared with C group. ^#^ p<0.01compared with D group. Statistical analyses were made using the one-way ANOVA followed by the Tukey’s test.

The results showed that the thiol content of hippocampus decreased in D group (p<0.001), DM group (p<0.01), and DE*-* 400 (p<0.001) compared to C group. Thiol concentration of hippocampus was significantly increased by Nigella sativa extract 200 mg/kg (p<0.05) in comparison to untreated diabetic group (Figure 2).

**Figure 3:**
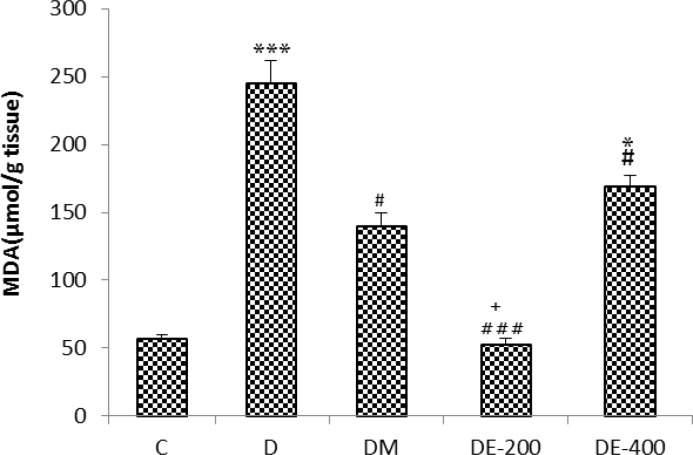
The Effect of Nigella sativa extract on the malondialdehyde (MDA) level in the hippocampus of the STZ-induced diabetic rats. n=8 in the untreated diabetic group and n=10 in all other groups. ^*^ p<0.05 and ^***^ p<0.001 compared with the C group.^ #^ p<0.01 and ^###^ p<0.001 compared with the D group.^ +^ p<0.05 comparison between DE-400 and DE-200 groups. Statistical analyses were made using the one-way ANOVA followed by the Tukey’s test.

In D group (p<0.001) and group DE-400 (p<0.05), the malondialdehyde (MDA) content of the hippocampus increased in comparison to the C group. However, malondialdehyde was reduced in the groups of DE-200 (p<0.001), DE-400 (p<0.05), and DM (p<0.05) in comparison to the D group (Figure 3).

**Table 1.** Effect of Nigella sativa seed extract on the average serum concentration of glucose (mg/dl) in the STZ-induced diabetic rats.

**Group**	**Day 0**	**Day 3**	**Day 24**	**Day 45**
**C**	86.10±2.62	86.10±2.62	87.38±4.31	84.48±3.96
**D**	82±2.11	335.2±17.54***	293.39±7.82***	300.54±10.40***
**DM**	98.46±1.67	302.43±10.32***	278.99±26.11***	267.60±21.31***
**DE-200**	91.91±5.89	347.33±69***	190.99±21.28*#×	168.75±24.32##×
**DE-400**	91.65±4.05	334.24±5.30***	258.74±28.72***	227.47±27.40*

Data are expressed as mean ± SEM. n=8 in the D group and n=10 in all other groups.

* p<0.05

*** p<0.001 compared to C group.

# p<0.01

## p<0.001 compared with the D group.

× p<0.05 comparison between DM and DE-200 groups. Statistical analyses were made using the one-way ANOVA followed by the Tukey’s test.

## Discussion

In this study, the ethanolic extract* of the Nigella Sativa*, 200 mg/kg, was effective in reducing the blood glucose level after 24 days (p=0.025), and this reduction persisted and was even more pronounced (p=0.003) until the day 45 without affecting the weight. 

It has been shown that hyperglycemia leads to elevated amount of the reactive oxygen species (ROS) (Maiese et al., 2007 ▶), and ROS plays a substantial role in the development of the diabetes mellitus and its complications (Maritim et al., 2003 ▶; Huber et al., 2006 ▶; Sani et al., 2012 ▶). Furthermore, hyperglycemia compromises the cerebral blood flow and brain glucose metabolism (Christopher et al., 2006 ▶).

*Nigella Sativa* has been reported to possess antidiabetic activity (Muhammad Tauseef Sultan et al., 2014 ▶). It has been reported that fixed, essential oil, methanolic extract, and commercial oil of *Nigella Sativa* stimulate the insulin secretion (Muhammad Tauseef Sultan et al., 2014 ▶; Houcher et al., 2007 ▶) as well as the hepatic alterations of the enzymes of the gluconeogenesis pathway (Houcher et al., 2007 ▶). Increasing of the insulin resistance in peripheral tissues (Samane, 2006 ▶), inhibition of glucose absorption in small intestine (Meddah, 2009 ▶), reduction of AGE (advanced glycation end-products) accumulation (Fararh, 2004 ▶), activation of the AMPK (AMP-activated protein kinase) pathway, and the increased expression of Muscle Glut4 (Benhaddou-Andaloussi, 2011 ▶) have also been demonstrated as mechanisms that Nigella sativa can reduce the blood glucose levels. Moreover, reduction of deteriorations of the pancreatic beta cell’s shape have been reported to occur as a result of the antioxidative properties of the *Nigella Sativa* management in STZ- diabetic rats (Ahmad et al, 2013 ▶).

So far, phenolic compounds especially flavonoids have been known to be responsible for antioxidative properties of the medicinal plants (Ramkissoon et al., 2013 ▶), and flavonoids have shown positive effects to decrease the blood glucose levels (Moradabadi et al., 2013 ▶). *Nigella Sativa *contains flavonoids (Al-okaily et al., 2012 ▶) and thymoquinone (TQ), the major effective component, (Alhebshi et al., 2013 ▶) that can restore the antioxidant levels to normal (Salem, 2005 ▶) and diminish blood glucose level (Badr G, 2011 ▶).

 In this study, different doses of the hydroalcoholic extract of the *Nigella Sativa* significantly decreased the amount of MDA in the hippocampus of the diabetic group, although the dose of 200 mg/ kg was more potent than 400 mg/kg. Furthermore, the thiol content of the hippocampus showed significant increase only with 200 mg/kg of the extract. It was shown that AGE increased in diabetes and caused an oxidative stress (Goh, 2008 ▶). Since the dosage of 200 mg/kg of Nigella sativa extract had the most beneficial effects on reducing the blood glucose concentration, low levels of glucose AGE levels may explain the higher thiol content in the current study. It has been reported that oral *Nigella Sativa* oil increased MDA level in the cortex of rats in an experimental model of oxidative stress (Neveen and Iman, 2010 ▶). However, the hydroalcoholic extract of the *Nigella Sativa* has been reported to increase the thiol content and reduce the MDA level of the cortex in rats with memory impairment (Hosseini et al., 2014 ▶). In this study, it seems that hydroalcoholic extract of *Nigella Sativa *at the dose of 200 mg/kg restored antioxidative capacity in the hippocampus of STZ-induced diabetic rats. Therefore, the clinical studies in diabetic patients can be suggested for future investigations to reveal any potential benefit of *Nigella Sativa* seeds to prevent cerebral complications of diabetes mellitus, especially memory impairments.

## Conclusion

*The Nigella Sativa* seeds extract showed antidiabetic and protective effects against the oxidative stress in the hippocampus of the STZ-induced diabetic rats.

## Conflict of Interests

The authors declare that there is no conflict of interest regarding the publication of this paper.
